# A Genome-wide study of blood pressure in African Americans accounting for gene-smoking interaction

**DOI:** 10.1038/srep18812

**Published:** 2016-01-11

**Authors:** Jacquelyn Y. Taylor, Karen Schwander, Sharon L. R. Kardia, Donna Arnett, Jingjing Liang, Steven C. Hunt, D.C. Rao, Yan V. Sun

**Affiliations:** 1 School of Nursing, Yale University, Orange; 2 Division of Biostatistics, School of Medicine, Washington University in St. Louis, St. Louis; 3 Department of Epidemiology, School of Public Health, University of Michigan, Ann Arbor; 4 Department of Epidemiology, School of Public Health, University of Alabama, Birmingham; 5 Department of Epidemiology and Biostatistics, School of Medicine, Case Western Reserve University, Cleveland; 6 Cardiovascular Genetics Division, School of Medicine, University of Utah, Salt Lake City.; 7 Department of Epidemiology, Rollins School of Public Health, Emory University; 8 Department of Biomedical Informatics, School of Medicine, Emory University, Atlanta

## Abstract

Cigarette smoking has been shown to be a health hazard. In addition to being considered a negative lifestyle behavior, studies have shown that cigarette smoking has been linked to genetic underpinnings of hypertension. Because African Americans have the highest incidence and prevalence of hypertension, we examined the joint effect of genetics and cigarette smoking on health among this understudied population. The sample included African Americans from the genome wide association studies of HyperGEN (N = 1083, discovery sample) and GENOA (N = 1427, replication sample), both part of the FBPP. Results suggested that 2 SNPs located on chromosomes 14 (*NEDD8*; rs11158609; raw p = 9.80 × 10^−9^, genomic control-adjusted p = 2.09 × 10^−7^) and 17 (*TTYH2*; rs8078051; raw p = 6.28 × 10^−8^, genomic control-adjusted p = 9.65 × 10^−7^) were associated with SBP including the genetic interaction with cigarette smoking. These two SNPs were not associated with SBP in a main genetic effect only model. This study advances knowledge in the area of main and joint effects of genetics and cigarette smoking on hypertension among African Americans and offers a model to the reader for assessing these risks. More research is required to determine how these genes play a role in expression of hypertension.

It has been widely accepted that environmental factors such as lifestyle behaviors that include excess tobacco usage can be hazardous to one’s health^1^,^2^. There is evidence that cigarette smoking is a risk factor for development of chronic diseases such as hypertension, stroke, chronic obstructive pulmonary disease, and end organ damage^1^,^2^,^3^,^4^. Both behavioral aspects of dependence of tobacco usage^3^,^4^ and genetic underpinnings^5^,^6^ have been independently noted in the literature to mediate and/or modulate overall health.

The term gene-environment (G × E) interaction is used to define the biological or statistical interplay between genetic and environmental factors^7^. The statistical interactions, defined as the non-additive effects of the gene and environment, have been widely investigated in genetic epidemiological studies. Using high-throughput genotyping technologies, it is possible to conduct genome-wide studies accounting for G × E interaction to identify genetic and environmental factors jointly influence a disease trait. However, challenges in study design and analytical methods remains in the GWAS era after decades of research in genetic epidemiology^7^,^8^. In simulation studies, a joint test for main genetic effect and interaction effects was recently proposed and showed improved power with the presence of moderate interaction effect or the interaction effect in the opposite direction of the main effect^9^. Manning *et al.* applied the joint test in a genome-wide study, and identified joint effects of SNP and SNP-BMI interaction related to fasting glycemic traits^10^.

Research in the area of genetics of cigarette smoking has expanded to include national consortiums^11^,^12^ and work groups for conducting genome wide association studies (GWAS) in this area^13^,^14^. However, none have considered the gene-environment interaction effects of cigarette smoking on predicting high blood pressure in an African American sample. We propose to examine African Americans participants in the Hypertension Genetic Epidemiology Network (HyperGEN) study, and replicate the association in African American participants from the Genetic Epidemiology Network of Arteriopathy (GENOA) study, two large epidemiological studies of hypertension genetics. We have selected African Americans as the target of our investigation because they have the highest incidence and prevalence of hypertension than any other ethnic group in the United States. Because African Americans are at greatest risk for development of hypertension^1^, it is important to consider the gene-environment interactions that may contribute to this disparity.

GWAS have paved the way for the examination of a multitude of single nucleotide polymorphisms (SNPs) that may contribute to development of various diseases such as hypertension^15^,^16^,^17^,^18^,^19^,^20^. The Cohorts for Heart and Aging Research in Genomic Epidemiology (CHARGE) Consortium identified genome-wide significant loci (p value < 5 × 10^−8^) for hypertension and systolic and diastolic blood pressure, respectively^15^. When combined with the Global Blood Pressure Genetics (GlobalBPGen) Consortium data^16^, the joint analysis of results identified 11 genome-wide significant associations with hypertension, systolic or diastolic blood pressure from 8 independent loci. Advancements made by GWAS aid in understanding the genetic underpinnings of blood pressure regulation and potential mechanistic interactions. The results of these consortium studies highlight the association between common variants, blood pressure, and hypertension and suggest potential target areas for cardiovascular disease prevention, intervention, and treatments. GWAS efforts have also found moderate associations with SNPs on genes found to be significant for high blood pressure among Europeans, but continued work must be done among African American populations^20^,^21^. The International Consortium for Blood Pressure Genome-wide Association Studies (ICBP-GWAS) found that blood pressure among African Americans is a trait with genetic foundations similar to those of European ancestry but with significant intricacy^20^. In a recent blood pressure GWAS of 29,378 individuals with African ancestry, five loci were genome-wide significant using transethnic analyses^22^. We propose to advance knowledge in this area of joint effect of genetics and cigarette smoking on blood pressure among African Americans. We offer a model to the reader for assessing genetic and smoking behavior risk main and interaction effects on predicting high blood pressure among African Americans.

## Results

Descriptive statistics of both the HyperGEN and GENOA samples can be found in table 1. We tested the association of the covariates with adjusted SBP and DBP in a full model including age, age^2^, sex, BMI, current Smoking and top ten principal components from the GWAS data (full model summarized in table 2). Age was consistently associated with both adjusted SBP and DBP. BMI and current smoking status were significantly associated with SBP but not DBP in the HyperGEN sample. Age^2^ and sex were only significantly associated with DBP in this African American sample. Three out of the top ten principal components were significantly associated with adjusted SBP and DBP.

**Figure 1:**
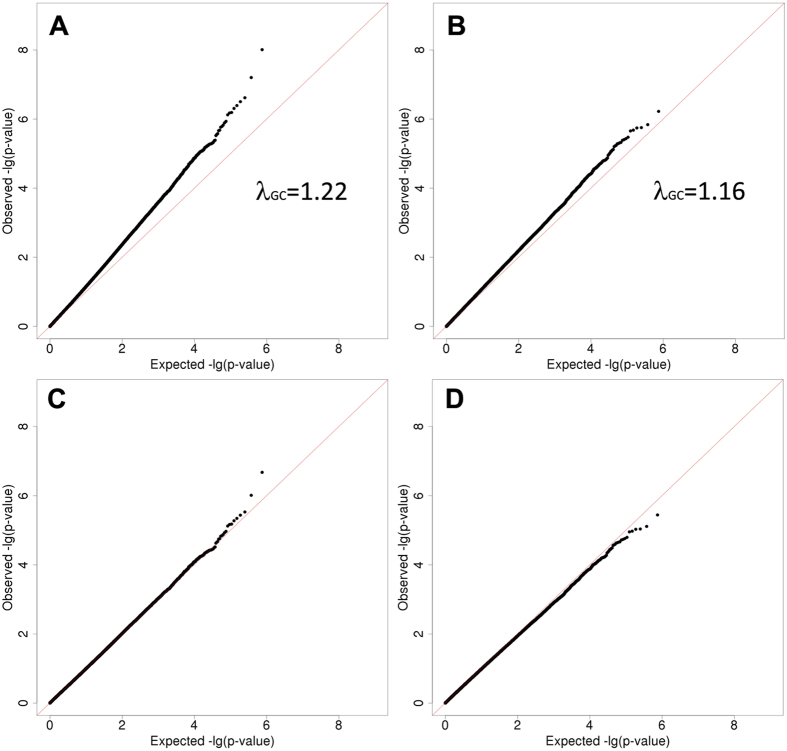
2 × 2 QQ Plot.

We conducted the joint analysis of the main SNP effect and the SNP × Smoking interaction of 761,050 SNPs in 1,083 African American participants of HyperGEN. We identified fourteen SNPs having significant joint association with SBP using a threshold of false discovery rate (FDR) q-value less than 0.1 (Table 3). Using a more conservative Bonferroni correction of multiple testing (nominal p-value of 6.57 × 10^−8^ of 761,050 SNPs), we identified two SNPs located on chromosome 14 (rs11158609) and 17 (rs8078051) significantly associated with SBP including the genetic interaction with cigarette smoking. SNP rs11158609 (p-value of 9.80 × 10^−9^, genomic control-adjusted p = 2.09 × 10^−7^) is located in the intronic region of neural precursor cell expressed, developmentally down-regulated 8 (*NEDD8*). SNP rs8078051 (p-value of 6.28 × 10^−8^, genomic control-adjusted p = 9.65 × 10^−7^) is also located in the intronic region of gene tweety homolog 2 Drosophila (*TTYH2)*. The QQ-plots (Fig. 1) of the p-values from the joint analysis suggested a moderate inflation of the genome-wide analysis. The inflation factors were 1.22 and 1.16 for SBP and DBP correspondingly, based on the method of genomic control^23^. After applying genomic control, the adjusted p-values of the most significant SNPs rs11158609 and rs8078051 were 2.09 × 10^−7^ and 9.65 × 10^−7^ (Table 3), which were no longer genome-wide significant. The p-values of the main SNP effects (without the interaction term in the model) of rs11158609 and rs8078051 are 3.95 × 10^−5^ and 4.19 × 10^−3^ respectively. The p-values of SNP × Smoking term (1df test) are summarized in Supplementary table 1. For DBP, we did not identify any significant joint association including both main genetic effect and the SNP × Smoking interaction after correction for multiple testing. SNP rs11213629 with the lowest p-value (5.99 × 10^−7^) is located in an intergenic region on chromosome 11.

We examined the impact of alternative BP adjustment methods for antihypertensive use on the 2df SNP × Smoking interaction analysis in HyperGEN African Americans. In Supplementary table 2, we summarize the p-values of the top associations from three adjustment methods for antihypertensive use: 1) add category-specific mean to SBP/DBP; 2) add 10/5 mmHg to SBP/DBP; 3) add 15/10 mmHg to SBP/DBP for patients taking antihypertensive medications. The most significant associations from method 1 also show similar low p-values using method 2 and 3. Among fourteen top associations, method 1 provides the lowest p-values in thirteen SNPs. For instance, rs11158609 and rs8078051, the two most significant SNPs using method 1, have p-values of 1.08 × 10^−6^ and 4.71 × 10^−7^ using method 2, and 6.09 × 10^−7^ and 5.17 × 10^−7^ using method 3.

**Figure 2:**
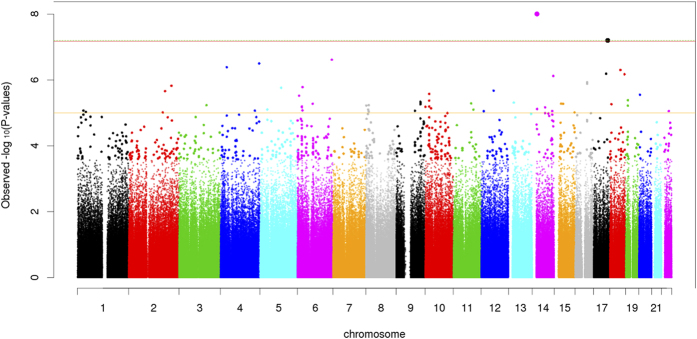
Systolic Blood Pressure (SBP) Manhattan Plot.

**Figure 3:**
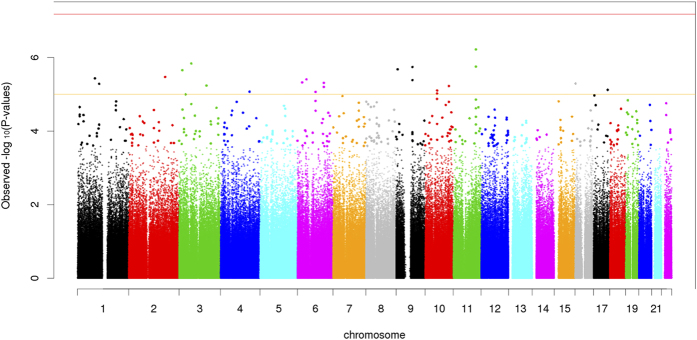
Diastolic Blood Pressure (DBP) Manhattan Plot.

Using 1,427 African American participants from the GENOA study, we did not replicate the fourteen top associations (FDR less than 0.1 in HyperGEN) with SBP or DBP by testing the joint effects of SNP and SNP × Smoking interaction, at the alpha level of 0.05 (Table 3). However, the joint association between rs11158609 and SBP remained significant (p-value of 2.98 × 10^−8^) in the combined test of HyperGEN and GENOA African American samples. Further illustration of these findings can be found in Figs 2,3 (Manhattan Plots of joint SNP × Smoking interaction associated with SBP and DBP).

We conducted additional tests for hypertension to compare the association results with SBP for the most significant SNPs.We further examined the association with hypertension status using the generalized estimating equation (GEE) models to adjust for the familial relatedness. Using the likelihood ratio test to compare the full model with SNP and SNPxSmoking interaction terms and the reduced model without these two terms, we examined the joint effects of top two SNPs, rs11158609 and rs8078051. In both cases, the SNP terms improve the fitting with p-values of 0.12 and 0.0078 for rs11158609 and rs8078051 respectively, which is consistent with the association with SBP with less degree of significance.

## Discussion

The two most significant loci associated with SBP are located in two known genes, *NEDD8* and *TTYH2* respectively. Interestingly, the protein products of *NEDD8* and *TTYH2* were both predicted to bind with neural precursor cell expressed, developmentally down-regulated 4-like, E3 ubiquitin protein ligase (*NEDD4L*) with moderate confidence scores of 0.589 (*TTYH2*) and 0.281 (*NEDD8*) using Search Tool for the Retrieval of Interacting Genes/Proteins (STRING) 9.0^24^, an online database of known and predicated protein-protein interaction. Although cigarette smoking is a major risk factor for hypertension, it is also widely accepted as a risk factor for cancer, therefore, we will discuss the genetic links with both hypertension and briefly with cancer.

### TTHY2

Tweety proteins are a family of chloride ion channels located extracellularly with the carboxyl-terminal domain located cytoplasmically^25^,^26^and in humans is thought to function as a volume-regulated chloride channel^25^,^27^,^28^,^29^. Reference alleles are G/A, the ancestral allele being G, and MAF is 0.04^29^. Located at 17q24, the population genetics for African Americans for the G allele is 84% and for the A allele is 16%^28^. NEDD4-2 (*NEDD4L*) was found to mediate ubiquitination of the tweety family members, in particular ion channels^25^. This is significant in that TTHY2 may function in addition to ion channel activity within mediating cell-to-cell interactions by binding to integrins. *NEDD4L* gene encodes a member of the NEDD4 family of HECT domain E3 ubiquitin ligases, which target specific proteins for lysosomal degradation. The NEDD4L protein mediates the ubiquitination of multiple target substrates and plays a critical role in epithelial sodium transport by regulating the expression of the epithelial sodium channel. SNP in *NEDD4L* is suggested to be associated with essential hypertension^30^. Genetic association studies have identified that *NEDD4L* variants were associated with blood pressure response to diuretics^31^,^32^,^33^. In addition, a SNP close to *NEDD4L* is strongly associated with the serum level of a liver enzyme, gamma-glutamyl transferase (p-value of 3 × 10^−12^) in a GWAS^34^. In other genomic studies, NEDD4L has also been shown to be associated with hypertension^35^, obesity^36^, smoking and cancer^37^ among a homogeneous population of Chinese Kazakh herders. Although these informative studies shed light on the associations of NEDD4L and hypertension and smoking independently, our study expands this work by examining and the independent and interactive effects of genetics and smoking on blood pressure among an African American population.

### NEDD8

NEDD8, also known as neddylin, a ubiquitin-like protein that functions in cell cycle control and embryogenesis. NEDD8 is highly expressed in heart, skeletal muscle, spleen, thymus, prostate, testis, ovary, colon, and in leukocytes^38^,^39^,^40^,^41^,^42^. *NEDD8* gene is located on chromosome 14 position 24688814, with SNP alleles A/G, the ancestral allele being A, and MAF is 0.108. The NEDD8 pathway has been shown to play an important part in regulating the actin cytoskeleton and cellular morphology^43^.

Specifically in strokes, after toxic levels of ischemia, an accumulation of ubiquitinated proteins have been found in damaged brain regions, while under conditions of mild or reversible ischemia, the ubiquitinated proteins have been shown to play a protective role^44^,^45^,^46^,^47^. In patients with antiphospholipid syndrome, an acquired autoimmune disorder of arterial or venous thrombosis and/or pregnancy morbidity in the presence of antiphopholipid antibodies, their was the presence of proteins NEDD8, RhoA, Hsp60, annexin I-II, and protein disulfide isomerase which are all related to procoagulant states^46^. While this research including a number of genes and proteins is still relatively new, the proteasome may be involved in gene expression, transcription, cell cycle regulation, apoptosis, and the inflammatory process involving a number of disease processes such as hypertension^41^,^48^.

### Approach and Comparison with Previous Findings

Our analysis approach does not test a “perfect” or “pure” smoking-SNP interaction. Several statistical methods have been developed to assess gene-environment interactions on a genome-wide scale^7^. Our primary goal here is to identify genetic loci associated with blood pressure traits considering the interaction effects. This approach can potentially improve power by combining both marginal and interaction effects with limited sample size^10^. Because this is a genetic association study of African Americans, we examined the SBP-associated SNPs reported from the COGENT (African Ancestry) blood pressure GWAS in our cohort^22^. Both HyperGEN and GENOA contributed to the COGENT BP GWAS study. There are two SNPs associated with SBP with p-value less than 10^−6^, rs11041530 (*CYB5R2*) with p-value of 4.0 × 10^−8^ and rs17428471 (*EVX1-HOXA*) with p-value of 4.0 × 10^−7^. Although the SNP main effects of rs11041530 are similar (−1.67 in HyperGEN and -1.35 in COGENT), the association is not significant (p-value of 0.28) and may be due to limited sample size. Using the SNAP database^49^, we did not identify any proxy SNPs of rs17428471 on the Affymetrix 6.0 array with R^2^ > 0.8 in YRI. For the top two associations from the joint test, rs11158609 (NEDD8) and rs8078051 (TTYH2), we looked up their main genetic effects in the COGENT BP meta-analysis^22^. Neither SNPs were significantly associated with SBP nor DBP (p-value > 0.05) with inconsistent direction of additive genetic effects across nineteen participating cohorts.

### Limitations

Some limitations of the present study need to be considered. The top two SNPs have unusually large effect size. The joint SNP and SNP × Smoking interaction effects explain about 3% of variance (i.e. R^2^) of SBP based on the partial F-test. In previous GWAS of blood pressure readings, the identified SNP associations have much smaller effect sizes. For example, the top 29 blood pressure-associated SNPs identified by the ICBP collectively explains only 0.9% of the phenotypic variance for SBP^20^. Assuming effect size of 2% R^2^ for the joint test, we have 22% power using the current AA sample, and only 1.6% power for detecting joint effect size of 1% R^2^. Thus, having larger sample size of consistently phenotyped AAs will be critical for discovering BP-associated SNPs and genes in the future, in addition to the implementation of complementary statistical approaches.

The approach was based on the premise that susceptibility alleles for common diseases were not under strong negative selection, and common variants contributed to common disease traits (i.e. the ‘common disease-common variant’ hypothesis)^50^. However, the allelic spectrum for genes associated with complex quantitative traits, such as BP, was not fully delineated. It was possible that multiple rare polymorphisms in the biological and positional candidate genes that were studied could influence BP. Due to a lack of statistical power, identifying associations with BP using such alleles would not be possible using approaches employed in this study. The inferences may not be generalizable to individuals who are younger, normotensive, or of other ethnic groups. The validation of the Dinamap sphygmomanometers used in the HyperGEN study to collect blood pressure readings has been accepted for clinical investigation worldwide. In terms of GENOA, the use of random-zero sphygmomanometers for measuring blood pressure have been validated for many years. Despite some limitations, the approach employed in the present study illustrated the use of SNPs in candidate genes to construct a more complete picture of the genetic architecture of complex traits, such as BP.

Genomic control (GC) is an effective tool to control for type I error in GWAS. However, it tends to over-correct the most significant results and reduces power (i.e. increase type II error). Therefore, we presented both the unadjusted and GC-adjusted results to balance the strength and limitation of GC. To account for population stratification, we adjusted for the top 10 PCs in all regression models. No inflation was observed in the genome-wide SNP association analysis. Thus, the observed inflation is unlikely due to population stratification.

We observed modest inflation of low p-values (inflation factor of 1.22 and 1.16 for SBP and DBP respectively) from the 2df joint test for SNP × Smoking interaction (Fig. 1A,B). The p-values are less significant after control for the inflation (Fig. 1C,D). Furthermore, we recognize that replication is important for validation of genetic effects. Insufficient sample sizes (full sample and re-sampled subsets) or random measurement error may have limited the power to detect the effects of SNP × Smoking interaction. Additional replication with independent African American cohorts with similar characteristics are necessary to further validate the findings.

Genome-wide association is a favored approach for genetic studies of common human diseases. However, localizing and identifying genes underlying environmental variations and the occurrence of high blood pressure poses a formidable challenge. The next step in studying African Americans and high blood pressure could use a similar research design incorporating a genome-wide association and other approaches in studying gene-cigarette smoking interactions on high blood pressure.

## Conclusions

Many of the genetic studies utilizing data to uncover genes or proteins responsible for underlying disease states utilize genome wide association studies with multivariate analysis and pairwise combinations of traits, specifically addressing genes that serve in a pleiotropic role^51^. This enables phenotype and genotype information to be reflected in correlation to a combination of traits. This is a useful approach in that many unique variants within a number of genes can be studied in association with disease traits. Though many genetic determinants have been found significantly associated with hypertension^52^,^53^ and/or metabolic syndrome for instance^54^, further research is indicated to understand the protein-gene interaction, methylation patterns, and signaling pathways, which if studied could provide novel approaches to pharmacologic care, genetic testing, and to combating disease. *TTYH2* and *NEDD8* may play a role in the genetic architecture of cardiovascular disease, blood pressure regulation, and other disorders. Further research is indicated for a better understanding of how these genes play a role in expression of hypertension.

## Methods

The parent study, the GENOA and HyperGEN both had HIC/IRB approval via University of Mississippi Medical Center, University of Michigan and Washington University in St Louis and the methods were carried out in accordance with the approved guidelines. Each study performed its own quality control procedures centrally, and shared the cleaned genotypic data for GWAS analysis. Therefore, the SNP inclusion/exclusion criteria between the HyperGEN and GENOA studies are not completely identical and details of both are provided.

## Study Sample—HyperGEN

Genetic data for HyperGEN were completed utilizing the Affymetrix Genome-Wide Human SNP Arrays. Subjects with a call rate below 95% or with a sex mismatch were excluded. In HyperGEN, the majority of African Americans were genotyped with the Affymetrix Genome-Wide Human SNP Array 6.0. We removed samples with sex-mismatch and pedigree errors. We also removed monomorphic SNPs and SNPs with missing rate >5% or Hardy-Weinberg p-value < 10^−6^, and removed any genotypes with a non-Mendelian pattern of inheritance^21^,^22^,^55^. The final dataset for the GWAS analysis of blood pressure includes 761,050 SNPs in 1,083 African American participants. (See Table 1 for HyperGen demographics).

### The Genetic Epidemiology Network of Arteriopathy (GENOA)

Participants in GENOA who self-reported their ethnicity as African-American were included in the analyses for the present study. Participant samples were genotyped for 906,602 SNPs using the Affymetrix^®^ Genome-Wide Human SNP Array 6.0 platform. Participants were excluded if they had an overall SNP call rate <95% or sex mismatch between genotypic and phenotypic measurement. SNPs were excluded if they had unknown chromosomal location, a call rate less than 95% or a minor allele frequency (MAF) less than 0.05. After quality control filters, 650,367 SNPs were available for analysis in 1,427 GENOA African American participants who had both genotype and phenotype data.

### Phenotype Measurement

A clinical visit was performed and each subject was interviewed by study personnel for data collection for all variables under study. Blood pressure was obtained using digitized blood pressure sphygmomanometers and was collected in accordance with JNC-7 guidelines. In the analyses, we corrected for anithypertension medications among the HyperGEN participants in accordance with designations outlined by HyperGEN investigators which can be found in full detail in Wu *et al.* 2005^56^. To compare the impact of various adjustment methods of antihypertensive use on SNP × Smoking interaction analysis, we conducted sensitivity analysis of two alternative BP adjustment methods by adding 10/5 mmHg (CHARGE BP GWAS)^15^ or 15/10 mmHg (GlobalBPGen GWAS)^16^ to SBP/DBP for subjects taking antihypertensive medications in HyperGEN African Americans. There are 56.8% HyperGEN participants taking antihypertensive medication.

For GENOA participants taking antihypertensive medication, we corrected SBP and DBP values by adding 10 and 5 mmHg correspondingly^57^,^58^. There are 63.1% GENOA participants taking antihypertensive medication. The operational definition for hypertension in the GENOA study was described as SBP ≥ 140 or DBP ≥90 mm Hg or antihypertension drug prescription at the clinical assessment interview^22^. Height and weight were measured using standard clinical methods and body mass index (BMI) was calculated using CDC guidelines for adults^55^,^59^.

In the GENOA study, the status of cigarette smoking was collected from a self-reported questionnaire as “smoked within the past year”, “not smoked within the past year”, “never smoked”. The variable “current smoker” was defined as an individual who smoked within the past year^60^. For HyperGEN, cigarette smoking was measured similarly, with participants being asked if they “ever smoked cigarettes” or if they “smoke cigarettes now” to indicate if they were current smokers and (for current smokers and past smokers) “Number of cigarettes smoked per day”^55^.

Age, sex and other phenotypic data were collected from the physical examination and laboratory assessment at the time of the Phase I study visit in GENOA. The GENOA and HyperGEN studies were approved by the Institutional Review Boards of all participating institutions: University of Mississippi Medical Center, University of Michigan and Washington University in St Louis. Each participant gave written informed consent.

## Statistical Analysis

We used linear mixed models^61^ to test for association between each SNP and the medication-adjusted phenotypes (SBP/DBP) in order to account for family structure within HyperGEN (siblings) and GENOA. Each SNP was tested for additive effects in association with the outcome of interest in a test with one degree of freedom. We also tested the SNP×smoking interaction effects of medication-adjusted SBP and DBP using multivariable linear mixed models with age, age^2^, BMI and top 10 principal components (PCs) of the GWAS data, as covariates. For participants who were taking antihypertensive medication we corrected the observed SBP and DBP values by adding the mean effect of each of the six major antihypertensive medication categories which include: angiotensin-converting enzyme (ACE) inhibitors, alpha1-blockers, cardioselective beta-blockers (beta1-blockers), calcium channel blockers, thiazide and thiazide-like diuretics, and loop diuretics. Data management, descriptive statistics for the covariates and outcome variables, and the regression analyses were conducted using the statistics software package R in version 2.11.1 (http://www.r-project.org/).

Several methods to study Gene × Environment (G × E) interactions are available. In this study, we applied the joint analysis approach^9^ to identify genetic variants associated with BPs considering the interaction between SNP and cigarette smoking. This approach can examine both the main genetic effect and the G × E interactions in a single analysis^10^. We implemented this approach in our study using the following regression models.


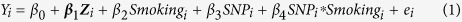


Where *Y*_*i*_ is the BP outcome (e.g. SBP and DBP) for person i, ***Z***_***i***_ is a vector of adjustment variables including age, age^2^, sex, BMI and the first 10 PCs of the common genome-wide SNPs, *Smoking*_*i*_ is the smoking status of person i, and *SNP*_*i*_ is the additive genetic effect of a SNP. *β*_*4*_ represents the effect of SNP × Smoking interaction.


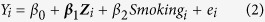


The full model (1) included SNP, smoking and SNP × Smoking terms, and the reduced model (2) only included smoking. We used a 2 degree-of-freedom (*df*) partial F-test to examine the null hypothesis H_0_: beta SNP = beta SNP×Smoking = 0. This joint test of SNP main effect and SNP × Smoking interaction allowed for screening the genetic effect conditioning on smoking, and the SNP × Smoking interaction effect simultaneously. In the discovery stage, we examined 761,050 SNPs (after quality control) in 1,083 HyperGEN African American participants.

In HyperGEN African Americans, to test the main SNP effect as a comparison, we conducted the standard GWAS of the same SNPs using model (3), which did not include the interaction term as in model (1). In all genetic analyses, appropriate methods will be used to account for multiple testing (see details below).


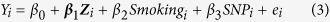


Using 1,427 African Americans from GENOA study measured by Affymetrix 6.0 array, we examined the most significant joint associations of SNP and cigarette smoking with adjusted SBP and DBP to replicate the findings in HyperGEN African Americans. After applying the same static tests by contracting model (1) and (2), we combined the p-values of the same SNP from the two samples using Fisher’s product method: 
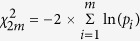
 where m is the number of p-values, *p*_*i*_ is the p-value of the i^th^ hypothesis test, and the chi-square distribution has 2m degrees-of-freedom, under the null hypothesis.

## Additional Information

**How to cite this article**: Taylor, J. Y. *et al.* A Genome-wide study of blood pressure in African Americans accounting for gene-smoking interaction. *Sci. Rep.* **6**, 18812; doi: 10.1038/srep18812 (2016).

## Supplementary Material

Supplementary Information

## Figures and Tables

**Table 1:** Descriptive statistics of the HyperGEN sample.

Variable	HyperGEN			GENOA		
	Pooled(n = 1083)Mean ± SD	Male(n = 354)Mean ± SD	Female(n = 729)Mean ± SD	Pooled(n = 1427)Mean ± SD	Male(n = 438)Mean ± SD	Female(n = 989)Mean ± SD
Age (year)	44.07 ± 13.38	42.86 ± 13.64	44.66 ± 13.21	58.64 ± 9.96	59.84 ± 9.58	58.12 ± 10.07
BMI (kg/m^2^)	32.57 ± 8.19	29.82 ± 6.55	33.91 ± 8.57	31.09 ± 6.65	28.47 ± 4.96	32.22 ± 6.95
Waist circumference (cm)	102.88 ± 18.96	100.17 ± 17.48	104.18 ± 19.51	103.36 ± 16.74	100.66 ± 12.73	104.52 ± 18.07
Hip circumference (cm)	114.68 ± 16.44	108.31 ± 13.39	117.77 ± 16.90	113.26 ± 14.67	105.72 ± 11.00	116.49 ± 14.87
Waist-hip-ratio	0.89 ± 0.08	0.92 ± 0.08	0.88 ± 0.08	0.91 ± 0.08	0.95 ± 0.05	0.89 ± 0.08
SBP (mmHg)	129.08 ± 22.24	129.93 ± 21.29	128.67 ± 22.69	136.41 ± 22.96	135.32 ± 22.20	136.88 ± 23.27
DBP (mmHg)	73.90 ± 11.73	76.50 ± 12.61	72.64 ± 11.08	77.49 ± 12.25	79.22 ± 12.50	76.75 ± 12.08
Adjusted SBP (mmHg)	142.84 ± 28.85	142.06 ± 27.91	143.22 ± 29.31	142.72 ± 24.39	140.90 ± 23.61	143.50 ± 24.68
Adjusted DBP (mmHg)	82.77 ± 15.29	84.26 ± 16.61	82.05 ± 14.57	80.65 ± 12.55	82.01 ± 12.72	80.06 ± 12.43
Cholesterol (mg/dL)	191.53 ± 41.58	186.29 ± 40.32	194.09 ± 41.97	204.97 ± 46.11	198.11 ± 47.86	207.91 ± 45.04
HDL cholesterol (mg/dL)	53.27 ± 15.31	48.73 ± 13.97	55.49 ± 15.45	55.46 ± 18.01	49.50 ± 17.52	58.01 ± 17.61
Triglycerides (mg/dL)	107.03 ± 85.30	113.34 ± 89.01	103.94 ± 83.31	146.69 ± 90.32	150.59 ± 116.75	145.01 ± 76.25
						
Ever Smoker	48.5%	63.6%	41.2%	43.3%	67.7%	32.9%
Current Smoker	28.5%	38.1%	23.9%	19.5%	28.8%	15.6%
Diabetes	19.4%	15.8%	21.1%	22.3%	19.0%	23.8%
Hypertension	68.7%	62.4%	71.8%	73.8%	69.8%	75.5%
						

**Table 2:** The associations of covariates and systolic and diastolic blood pressure.

Variables	Adjusted SBP			Adjusted DBP		
	Beta coefficient	SE	P-value	Beta coefficient	SE	P-value
Age	1.75	0.320	< 0.001	2.11	0.173	< 0.001
Age^2^	−6.87 × 10^−3^	3.53 × 10^−3^	0.051	−1.76 × 10^−2^	1.91 × 10^−3^	< 0.001
Sex	−1.93	1.57	0.218	−2.44	0.842	0.004
BMI	0.662	9.32 × 10^−2^	< 0.001	−1.35 × 10−^2^	5.04 × 10−^2^	0.788
Current Smoking	5.73	1.65	< 0.001	1.66	0.889	0.062
PC1	35.6	26.5	0.181	30.2	14.8	0.042
PC2	−43.0	33.2	0.196	−9.04	19.5	0.644
PC3	69.2	29.3	0.019	28.1	16.8	0.094
PC4	−29.3	30.8	0.342	−3.74	17.9	0.835
PC5	−6.78	29.5	0.819	−8.15	16.9	0.630
PC6	−27.3	31.1	0.380	−2.43	18.1	0.893
PC7	23.0	32.8	0.484	31.6	19.2	0.099
PC8	−65.4	32.0	0.041	−38.1	18.6	0.041
PC9	1.78 × 10^−2^	31.0	1.00	24.9	18.0	0.166
PC10	−72.7	29.9	0.015	−49.7	17.2	0.004

**Table 3:** Joint Association (2df) of SNP main effect and SNP × Smoking interaction with SBP with FDR q-value less than 0.1.

SNP	Chr	Position(GRCh37/hg19)	Band	Allele#	MAF	ClosestGene	Location	HyperGENP-value	HyperGENP_GC_	HyperGENFDR	GENOAP-value	CombinedP-value
rs11158609	14	24688814	q12	G/A	0.079	*NEDD8*	intronic	9.80 × 10^−9^	2.09 × 10^−7^	0.007	0.142	2.98 × 10^−8^
rs8078051	17	72251240	q25.1	G/A	0.142	*TTYH2*	intronic	6.28 × 10^−8^	9.65 × 10^−7^	0.024	0.770	8.63 × 10^−7^
rs3817779	6	166905534	q27	T/C	0.136	*RPS6KA2*	intronic	2.42 × 10^−7^	2.94 × 10^−6^	0.059	NA	NA
rs11726022	4	188170467	q35.2	G/A	0.149	LOC339975*	intergenic	3.14 × 10^−7^	3.64 × 10^−6^	0.059	0.381	2.03 × 10^−6^
rs1596724	4	32555853	p15.1	T/C	0.107	LOC102723828*	intergenic	4.06 × 10^−7^	4.50 × 10^−6^	0.061	0.170	1.21 × 10^−6^
rs1792738	18	53845164	q21.31	G/A	0.091	LINC01539*	intergenic	4.93 × 10^−7^	5.29 × 10^−6^	0.062	0.361	2.94 × 10^−6^
rs8065772	17	64842925	q24.2	T/C	0.126	*CACNG5*	intronic	6.44 × 10^−7^	6.59 × 10^−6^	0.063	0.136	1.51 × 10^−6^
rs1157477	18	73850510	q23	G/A	0.200	*LOC339298*	intronic	6.66 × 10^−7^	6.78 × 10^−6^	0.063	0.315	3.43 × 10^−6^
rs4906269	14	103362017	q32.32	C/A	0.389	*TRAF3*	intronic	7.53 × 10^−7^	7.50 × 10^−6^	0.063	NA	NA
rs1862746	16	60462358	q21	C/A	0.413	LOC729159*	intergenic	1.16 × 10^−6^	1.07 × 10^−5^	0.088	0.201	3.80 × 10^−6^
rs1862745	16	60462336	q21	T/C	0.416	LOC729159*	intergenic	1.29 × 10^−6^	1.17 × 10^−5^	0.089	0.429	8.52 × 10^−6^
rs1012264	2	208115003	q33.3	G/A	0.346	*LOC101927865*	intronic	1.50 × 10^−6^	1.33 × 10^−5^	0.093	0.914	1.99 × 10^−5^
rs1028885	6	25434518	p22.2	G/A	0.421	*LRRC16A*	intronic	1.64 × 10^−6^	1.43 × 10^−5^	0.093	0.530	1.30 × 10^−5^
rs639709	5	103311439	q21.2	G/C	0.153	NUDT12*	intergenic	1.72 × 10^−6^	1.48 × 10^−5^	0.093	0.976	2.40 × 10^−5^

P_GC_: P-values adjusted for genome-wide inflation using genomic control (GC).

^#^the first allele is the effect allele, and the second allele is the reference allele.

^*^the closest gene within 1 Mbp flanking region of the SNP.
