# Supplementary material for: A Genome-wide study of blood pressure in African Americans accounting for gene-smoking interaction
Source: Sci Rep. 2016 Jan 11;6:18812. doi: 10.1038/srep18812 (PMC4707536; doi:10.1038/srep18812)
Supplement: Supplementary Information [file srep18812-s1.pdf]

A Genome-wide study of blood pressure in African Americans

accounting for gene-smoking interaction

Authors: Jacquelyn Y. Taylor[a], Karen Schwander[b], Sharon L. R. Kardia[c], Donna Arnett[d], The COGENT BP Consortium[e], Steven C. Hunt[f], D.C. Rao[b], Yan V. Sun[g,h]

[a]School of Nursing, Yale University, Orange; [b]Division of Biostatistics, School of Medicine, Washington University in St. Louis, St. Louis; [c]Department of Epidemiology, School of Public Health, University of Michigan, Ann Arbor; [d]Department of Epidemiology, School of Public Health, University of Alabama, Birmingham; [e]The COGENT BP Consortium; [f]Cardiovascular Genetics Division, School of Medicine, University of Utah, Salt Lake City; [b]Division of Biostatistics, School of Medicine, Washington University in St. Louis, St. Louis; [g]Department of Epidemiology, Rollins School of Public Health, Emory University; [h]Department of Biomedical Informatics, School of Medicine, Emory University, Atlanta

The members of the COGENT BP Consortium are Nora Franceschini, Ervin Fox, Zhaogong Zhang, Todd L. Edwards, Michael A. Nalls, Yun Ju Sung, Bamidele O. Tayo, Yan V. Sun, Omri Gottesman, Adebawole Adeyemo, Andrew D. Johnson, J. Hunter Young, Ken Rice, Qing Duan, Fang Chen, Yun Li, Hua Tang, Myriam Fornage, Keith L. Keene, Jeanette S. Andrews, Jennifer A. Smith, Jessica D. Faul, Zhang Guangfa, Wei Guo, Yu Liu, Sarah S. Murray, Solomon K. Musani, Sathanur Srinivasan, Digna R. Velez Edwards, Heming Wang, Lewis C. Becker, Pascal Bovet, Murielle Bochud, Ulrich Broeckel, Michel Burnier, Cara Carty, Wei-Min Chen, Guanjie Chen, Wei Chen, Jingzhong Ding, Albert W. Dreisbach, Michele K. Evans, Xiuqing Guo, Melissa E. Garcia, Rich Jensen, Margaux F. Keller, Guillaume Lettre, Vaneet Lotay, Lisa W. Martin, Alanna C. Morrison, Thomas H. Mosley, Adesola Ogunniyi, Walter Palmas, George Papanicolaou, Alan Penman, Joseph F. Polak, Paul M. Ridker, Babatunde Salako, Andrew B. Singleton, Daniel Shriner, Kent D. Taylor, Ramachandran Vasan, Kerri Wiggins, Scott M. Williams, Lisa R. Yanek, Wei Zhao, Alan B. Zonderman, Diane M. Becker, Gerald Berenson, Eric Boerwinkle, Erwin Bottinger, Mary Cushman, Charles Eaton, Gerardo Heiss, Joel N. Hirschhorn, Virginia J. Howard, Matthew B. Lanktree, Kiang Liu, Yongmei Liu, Ruth Loos, Karen Margolis, Bruce M. Psaty, Nicholas J. Schork, David R. Weir, Charles N. Rotimi, Michele M. Sale, Tamara Harris, Sharon L.R. Kardia, Steven C. Hunt, Donna Arnett, Susan Redline, Richard S. Cooper, Neil Risch, D.C. Rao, Jerome I. Rotter, Aravinda Chakravarti, Alex P. Reiner, Daniel Levy, Brendan J. Keating, and Xiaofeng Zhu.

Short Title: Genetic-Smoking Interaction on Blood Pressure

Word count, manuscript: 4,053

Word count, abstract: 187

Figures: 3

Tables: 3

Supplementary Tables: 2

Disclaimers: None

Name and address of author(s) responsible for correspondence and to who requests for reprints should be made:

Jacquelyn Y. TAYLOR, PhD, PNP-BC, RN, FAAN
Address: Yale University, School of Nursing, 400 West Campus Drive
Orange, Connecticut, 06516-7399
Phone: 203-737-2364
Fax: 203-737-2364
Email: Jacquelyn.taylor@yale.edu

Yan V. SUN, Ph.D.
Department of Epidemiology and Biomedical Informatics, Emory University
1518 Clifton Road NE #3049
Atlanta, GA 30322
Phone: (404) 727-9090
Fax: (404) 727-8737
Email address: yvsun@emory.edu

Supplementary Table 1. The summary of SNP main effects and SNP×Smoking interaction effects in HyperGEN and GENOA AAs

SNP	Chr	Position (GRCh37/hg19)	Band	Allele[#]	MAF	Overlapping Gene	Location	HyperGEN SNP beta coefficient	HyperGEN SNP P-value	GENOA SNP beta coefficient	GENOA SNP P-value	HyperGEN P SNP×Smoking	GENOA P SNP×Smoking
rs11158609	14	24688814	q12	G/A	0.079	*NEDD8*	intronic	-8.12	3.95×10^{-5}	-0.09	0.954	1.20×10^{-5}	0.050
rs8078051	17	72251240	q25.1	G/A	0.142	*TTYH2*	intronic	4.39	4.19×10^{-3}	0.88	0.480	8.50×10^{-7}	0.890
rs3817779	6	166905534	q27	T/C	0.136	*RPS6KA2*	intronic	-1.54	0.287	NA	NA	8.92×10^{-8}	NA
rs11726022	4	188170467	q35.2	G/A	0.149		intergenic	7.63	2.85×10^{-7}	-1.46	0.306	0.0830	0.355
rs1596724	4	32555853	p15.1	T/C	0.107		intergenic	-6.02	5.79×10^{-4}	2.28	0.085	3.77×10^{-5}	0.467
rs1792738	18	53845164	q21.31	G/A	0.091		intergenic	2.76	0.130	-1.84	0.241	3.23×10^{-7}	0.423
rs8065772	17	64842925	q24.2	T/C	0.126		intergenic	-5.23	6.52×10^{-5}	-0.81	0.451	5.37×10^{-4}	0.065
rs1157477	18	73850510	q23	G/A	0.200	*LOC339298*	intronic	4.44	5.37×10^{-3}	0.16	0.904	7.43×10^{-6}	0.132
rs4906269	14	103362017	q32.32	C/A	0.389	*TRAF3*	intronic	1.98	0.063	NA	NA	9.14×10^{-7}	NA
rs1862746	16	60462358	q21	C/A	0.413		intergenic	-2.71	0.013	-0.09	0.923	5.61×10^{-6}	0.076
rs1862745	16	60462336	q21	T/C	0.416		intergenic	-2.70	0.013	0.09	0.924	6.33×10^{-6}	0.197
rs1012264	2	208115003	q33.3	G/A	0.346		intergenic	4.49	1.24×10^{-4}	-0.36	0.703	6.56×10^{-4}	0.859
rs1028885	6	25434518	p22.2	G/A	0.421	*LRRC16A*	intronic	-6.25	1.94×10^{-5}	-0.27	0.836	5.12×10^{-3}	0.272
rs639709	5	103311439	q21.2	G/C	0.153		intergenic	4.19	8.39×10^{-5}	0.20	0.827	1.18×10^{-3}	0.991

[#]: the first allele is the effect allele, and the second allele is the reference allele.

SNP P-value: the P-value of the test for SNP main effect

P SNP×Smoking: the p-value of the 1df test for SNP×Smoking

Supplementary Table 2. The sensitivity analysis of the 2df joint test for SNP×Smoking interaction effects using alternative blood pressure adjustments for antihypertensive medications.

SNP	Band	Overlapping Gene	Location	HyperGEN $*P_{adj1}$	HyperGEN $*P_{adj2}$	HyperGEN $*P_{adj3}$
rs11158609	q12	*NEDD8*	intronic	9.80×10^{-9}	1.08×10^{-6}	6.09×10^{-7}
rs8078051	q25.1	*TTYH2*	intronic	6.28×10^{-8}	4.71×10^{-7}	5.17×10^{-7}
rs3817779	q27	*RPS6KA2*	intronic	2.42×10^{-7}	3.53×10^{-7}	3.29×10^{-7}
rs11726022	q35.2		intergenic	3.14×10^{-7}	7.44×10^{-7}	8.39×10^{-7}
rs1596724	p15.1		intergenic	4.06×10^{-7}	3.02×10^{-6}	7.31×10^{-6}
rs1792738	q21.31		intergenic	4.93×10^{-7}	1.03×10^{-6}	7.47×10^{-7}
rs8065772	q24.2		intergenic	6.44×10^{-7}	2.45×10^{-7}	3.27×10^{-7}
rs1157477	q23	*LOC339298*	intronic	6.66×10^{-7}	1.01×10^{-6}	8.15×10^{-7}
rs4906269	q32.32	*TRAF3*	intronic	7.53×10^{-7}	2.67×10^{-5}	2.61×10^{-5}
rs1862746	q21		intergenic	1.16×10^{-6}	1.16×10^{-5}	4.19×10^{-6}
rs1862745	q21		intergenic	1.29×10^{-6}	1.43×10^{-5}	4.66×10^{-6}
rs1012264	q33.3		intergenic	1.50×10^{-6}	7.78×10^{-6}	1.09×10^{-5}
rs1028885	p22.2	*LRRC16A*	intronic	1.64×10^{-6}	6.70×10^{-6}	6.26×10^{-6}
rs639709	q21.2		intergenic	1.72×10^{-6}	9.10×10^{-5}	4.33×10^{-5}

* P_{adj1}: for subjects taking antihypertensive medications, we added the mean effect of each drug category to SBP and DBP as described in the Methods section.

* P_{adj2}: for subjects taking antihypertensive medications, we added 10 and 5 mmHg to observed SBP and DBP respectively.

* P_{adj1}: for subjects taking antihypertensive medications, we added 15 and 10 mmHg to observed SBP and DBP respectively.
